# Cholera, Dysentery, and Fever, Pathologically and Practically Considered

**Published:** 1847-10-01

**Authors:** 


Cholera, Dysentery, and Fever, pathologically and practically considered; or the Nature, Causes, Connexion, and Treatment of these Diseases in all their Forms. By *Charles Searle*, M.D., late of the E. I. Company Madras Establishment, &c. &c. Pp. 120. London: Churchill, 1847.

Dr. Searle is surely a very fortunate man above his fellows; for his work has gained for itself a very wide circulation, and the prestige of a favourable reception, ere it yet appeared before the public, or passed through the ordeal of the press. "The Court of Directors of the East India Company, with their usually enlightened concern for the millions under their paternal dominion, and regard for the welfare of the many in their employ, have enabled me—with a liberality demanding this public acknowledgment—to present to each of you [the medical officers of their service—*Rev.*] a copy of this publication, which has for its chief object, the dissemination of improved principles of treating that scourge of India, and it has been said—'*opprobrium medicorum*'—the Cholera."

Emboldened by so flattering a compliment, the author " calls upon the press generally to aid him in its circulation, at the same time that he calls upon the profession and all mankind either to confute him in argument, or to exhibit by facts, why the practice enjoined and the treatment recommended should not be accepted as deserving universal adoption." Now, without regularly entering into the lists with so proud and confident an opponent,—the limits of our space would not allow us to do justice either to ourselves or to him—we shall do little more at present than select a few passages from his message, adding but a word or two of our own in the way of remark.

*Nature of Malaria.*—" Sulphuretted hydrogen, one of the offensive gases issuing from sewers, and a product of the decomposition of animal and vegetable substances, is so truly poisonous to the animal system, that a bird, or other small animal, exposed to an atmosphere containing but one fifteen-hundredth part of this gas, dies almost immediately from its effects; and a horse has been killed by exposing it to breathe an atmosphere containing but one-250th part. That malaria, which is a compound of this and some other gases equally noxious, developed by the decomposition of animal and vegetable substances, wherever they exist, may be truly affirmed to be a poison; and which, under certain conditions of the atmosphere and states of the system, will produce cholera; and under other circumstances of exposure to its influence will occasion typhus fever, dysentery, scarlet fever, erysipelas, rheumatic fever, influenza, or other modification of fever of a remitting type with local affection—either of the brain, lungs, or abdominal organs." P. 28.

In a previous page, 484, we have questioned the propriety of regarding sulphuretted hydrogen or any chemical gas whatsoever as a necessary ingredient in the malaria or miasm that produces fever, whether of a periodic or of a continued type. We know of no experiments or observations that at all warrant the idea, more especially in reference to Intermittent or Remittent fevers, and still less as respects Scarlet fever or Rheumatism. Dr. Searle does not seem to be quite satisfied himself as to the origin and morbific effects of malaria, if we may judge from the following remarks :—

" We must not," says he, " confine our ideas to the immediate or direct decomposition of such (animal or vegetable) substances, issuing from sewers, drains, and other foul sources. We must not, however, confine our ideas to the immediate or direct decomposition of such substances, or to the sources that I have mentioned exclusively; the exhalations from marshes, or the paddy grounds of India, from jungle or forest, as well as from the uncleanly persons of both men and animals, or the deteriorations of the air by respiration in crowded apartments, and imperfectly ventilated or confined situations, are quite equal, in certain conditions of the system, to produce the same effects, viz., cholera, or, as I have before said, under other circumstances, typhus, or remittent fever, these diseases, under ordinary circumstances, having the same common origin, and being intimately associated in character; and hence, the latter in India, and the former in Europe, frequently becomes the sequel of the former affection." P. 35.

Are we to understand from this that exhalations "from the uncleanly persons of both men and animals, or the deteriorations of the air by respiration in crowded apartments," ever produce a case of genuine Remittent fever? If this be the meaning of our author, we must entirely dispute the assertion, and call upon him for his proofs.

Among the causes of Epidemic Cholera is enumerated a thunder-storm, and its *modus operandi* is thus attempted to be explained analogically:

" A thunder-storm may also be considered such a cause, as evinced by its capability of addling or destroying the vital qualification of eggs; or arresting the process of fermentation, and souring beer, exposed in a bad cellar to its influence; or, as it is said sometimes to do, to kill the fish in a pond—effects which are known to succeed to a thunder-storm of even half-an-hour's duration. Now, as the vitality of fish is dependent upon certain chemical changes which are going on in the body of the amimal, which are common to the rest of the animal creation, and the incubation of the egg is dependent upon changes of a like character, and the fermentation of beer also dependent upon the same, and which, in either case, are effected by the agency of the oxygen of the air, in its combination with the carbon of the blood of the animal, or that of the white of the egg, or of the carbon of the sugar of the beer—we are naturally led to infer, seeing the same chemical process is going on in man's system, and upon which his life is dependent, that this process may be arrested or impaired by the same cause, or some analagously suddenly altered electrical condition of the atmosphere—and the disease, as a consequence, be induced, in persons predisposed by previous derangements of health, or subjected to a greater amount of exposure to the influencing cause, or possessing a greater susceptibility of system to be affected by it." P. 107.

In the Treatment of Cholera, Dr. Searle seems to regard *calomel* as an all but specific remedy, not only from its potent action on the hepatic functions, but also as a direct antidote to the morbific cause. His own words are—

"With respect to the first-mentioned indication (restoring the liver's function) universal experience testifies, that calomel has a direct and immediate exciting effect on the liver, increasing its secretion and the flow of bile into the bowels; and further, universal assent will be given by the profession to the fact, that it not only excites secretion of the bile, but all the secretions; and if it excite all the secretive organs, it must necessarily act generally upon the system, and excite all the functions, including those of the heart and brain. That it does so, thirty years' experience justifies me in confidently asserting, the pulse manifesting its exciting operation. And further, as it can only thus operate in admixture with the blood, into which it must be admitted by absorption from the stomach, it must of necessity operate, as it is a stimulant, as an antagonist agent also, in supercession of the depressing influence of the poisonous cause of the disease; and if this be the case, it is a remedy to which we might, under ordinary circumstances, apply the term specific in the cure of this disease; and, as the fruit of all my experience, I fearlessly aver, that it is as much so as it is possible any single remedy can be." P. 49.

So bent is he upon affecting the system with Mercury, that, when calomel appears to have little effect, he recommends a solution of corrosive sublimate to be given both by the mouth and in the way of injection—an eighth part of a grain every half-hour, or so, until the pulse improves and excitement becomes developed. He suggests also the trial of mercurial inhalation. "As the simplest mode of practising it, a tile, or brick, being made red hot, and put upon some sand in a dish, may be placed beneath the bed-clothes, and the patient, enclosing his head, may be allowed to breathe the vapour developed by throwing half a drachm of calomel, or red sulphate of mercury, on the heated object; or it may be inhaled from the tube of a funnel inverted over the dish. This might be repeated every hour or two, till amendment takes place."

The profession is not likely to have much confidence in the judgment of a writer who suggests such extravagances as these, nor in the sober sense or honest word of any one who pretends that the early and steady application of any means whatsoever will *in general,* far less *invariably,* prove successful, as Dr. Searle does not hesitate to assert, in the cure of so lethiferous a disease as Pestilential Cholera.

As to our author's views respecting Fever, that "will-o-the-wisp," as he calls it, and whose proteiform disguise has, in his opinion, so baffled the efforts of all writers to reveal its true nature until he took the subject in hand himself, it is far from being easy to make them out with any degree of precision. We must therefore beg our readers to consult his work for themselves, if they wish to know more about them.

